# Westward Spread of Highly Pathogenic Avian Influenza A(H7N9) Virus among Humans, China

**DOI:** 10.3201/eid2406.171135

**Published:** 2018-06

**Authors:** Qiqi Yang, Wei Shi, Lei Zhang, Yi Xu, Jing Xu, Shen Li, Junjun Zhang, Kan Hu, Chaofeng Ma, Xiang Zhao, Xiyan Li, Feng Liu, Xin Tong, Guogang Zhang, Pengbo Yu, Oliver G. Pybus, Huaiyu Tian

**Affiliations:** Beijing Normal University, Beijing, China (Q. Yang, X. Tong, H. Tian);; Shaanxi Provincial Centre for Disease Control and Prevention, Xi’an, China (W. Shi, L. Zhang, Y. Xu, J. Xu, S. Li, F. Liu, P. Yu);; Xianyang Centre for Disease Control and Prevention, Xianyang, China (J. Zhang);; Baoji Centre for Disease Control and Prevention, Baoji, China (K. Hu);; Xi’an Centre for Disease Control and Prevention, Xi’an (C. Ma);; Chinese Center for Disease Control and Prevention, Beijing (X. Zhao, X. Li);; Chinese Academy of Forestry, Beijing (G. Zhang); University of Oxford, Oxford, UK (O.G. Pybus)

**Keywords:** HPAI, H7N9, highly pathogenic, avian influenza, live poultry market, phylogenetic analysis, Shaanxi, Xianyang, Baoji, Xi’an, Yulin, Anhui, Fujian, Zhejiang, Jiangsu, Chongqing, Henan, Hunan, Hubei, Guangxi, Guangdong, Hong Kong, China, influenza, viruses, respiratory infections

## Abstract

We report infection of humans with highly pathogenic avian influenza A(H7N9) virus in Shaanxi, China, in May 2017. We obtained complete genomes for samples from 5 patients and from live poultry markets or farms in 4 cities. Results indicate that H7N9 is spreading westward from southern and eastern China.

Avian influenza A(H7N9) virus caused 5 waves of human infection in China from its emergence in 2013 (*1*) through May 17, 2017. During that period, 1,564 laboratory-confirmed human cases and 612 deaths were reported; about half occurred during the fifth wave (*2*). The fifth wave not only infected many more persons but also spread north; previous H7N9 outbreaks had been documented only in eastern and southern China. Moreover, viruses recently isolated from human case-patients in Guangdong Province (A/Guangdong/17SF003/2016 and A/Guangdong/17SF006/2017) in southern China have been confirmed as highly pathogenic avian influenza (HPAI) A(H7N9) viruses, on the basis of their molecular (*3*) and biologic (*4*) characteristics.

We report cases of human infection with H7N9 virus, including in 1 person who was infected with a highly pathogenic variant, in Shaanxi Province, western China, during April and May 2017. We obtained complete genome sequences of H7N9 viruses from 5 patients and from 21 environmental samples obtained from live poultry markets (LPMs) and from poultry farms in 4 cities.

## The Study

A case of HPAI H7N9 virus infection in a human from Shaanxi Province was identified in the city of Yulin on May 26, 2017. On day 1 of illness onset, fever and fatigue developed; after 2 days, the patient received medical attention. On day 4, the patient was admitted to an intensive care unit, and on day 6, HPAI H7N9 infection was laboratory confirmed. After treatment with oseltamivir, the patient recovered. An additional 4 cases of infection with low pathogenicity avian influenza (LPAI) A(H7N9) virus among humans were identified during April 30–May 13, 2017, in Shaanxi Province: 2 in Xianyang, 1 in Baoji, and 1 in the capital city, Xi’an. Recorded symptoms included fever (100%); cough and fatigue (75%); and productive cough, diarrhea, and shortness of breath (50%). Patients received medical attention at an average of 3.75 (range 1–5) days after illness onset and were admitted to a hospital at an average of 6 (range 4–8) days after onset. Samples were laboratory confirmed as H7N9 virus at an average of 9 (range 6–14) days after onset. Of the 5 patients, 4 were treated with oseltamivir; 2 of those died.

Respiratory tract specimens (throat swab or sputum sample) were collected from patients and transferred to the national laboratory at the Chinese Center for Disease Control and Prevention (China CDC) for confirmation. Viral RNA was extracted from each sample by using nucleic acid isolation magnetic beads, according to the manufacturer’s instructions (TianLong Science and Technology Co. Ltd, Xi’an, China). Avian influenza virus H7 and N9 gene segments were detected in the total RNA by using an H7N9 dual-channel Taqman probe reverse transcription PCR kit (BioPerfectus Co. Ltd., Taizhou, China).

During May 2017, we collected 496 environmental samples (poultry feces and poultry cage swabs from LPMs and poultry farms) from 4 cities (Xi’an, Baoji, Xianyang, and Yuli) in Shaanxi Province (Technical Appendix Table 1). A total of 14 samples from LPMs were positive for H7N9 virus.

We extracted total RNA from the patient respiratory specimens and from H7N9-positive environmental samples and sequenced avian influenza virus genomes by using next-generation sequencing on an Illumina MiSeq (Illumina Inc., Shanghai, China). Sequence comparisons showed that the viruses we isolated from 1 patient in Yulin and from the 15 environmental samples had the same polybasic (PB) amino acid sequence (PEVPKRKRTAR/GL) at the hemagglutinin (HA) cleavage site as that in previously confirmed HPAI H7N9 viruses (A/Guangdong/17SF003, A/Guangdong/17SF006, and A/Taiwan/1/2017) (*3*–*5*), indicating that the viruses we isolated are also HPAI viruses. In addition, the HPAI viruses we isolated appear to have inherited the HA amino acid mutation G186V from LPAI viruses and reverted to 226Q from 226L, as observed in previously confirmed HPAI H7N9 viruses (*4*–*6*). Because G186V and Q226L indicate an increased level of binding to human-type influenza receptors (*7*,*8*), the ability of the HPAI viruses we isolated here to infect humans is likely similar to that of previously reported HPAI H7N9 viruses. Furthermore, the HA genes of all Shaanxi HPAI H7N9 viruses we isolated were >99% identical to those of previously isolated HPAI H7N9 viruses.

**Figure 1:**
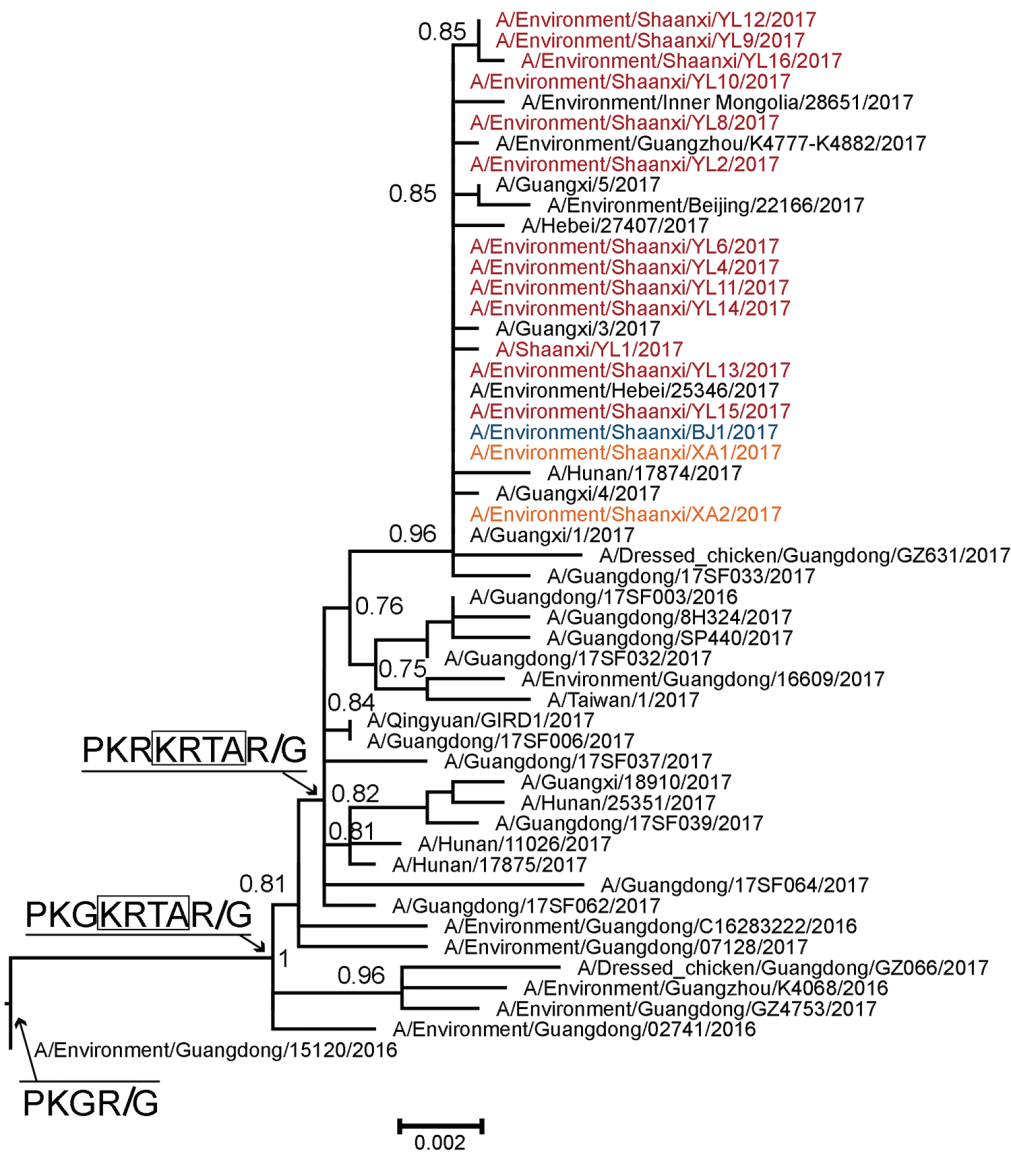
Detail of highly pathogenic avian influenza A(H7N9) viruses isolated from human and environmental sources, Shaanxi Province, China, 2016–2017, showing Shimodaira-Hasegawa-like local bootstrap support values. Amino acid changes within the hemagglutinin cleavage site are indicated on basal branches. The low pathogenicity strain A/Environment/Guangdong/15120/2016 is used as an outgroup. Colors indicate sampling locations of the H7N9 viruses obtained in this study: red, Yulin; blue, Baoji; orange, Xi’an. An expanded version of this figure showing comparisons to reference viruses is available in Technical Appendix Figure 1. Scale bar indicates amino acid substitutions per site.

**Figure 2:**
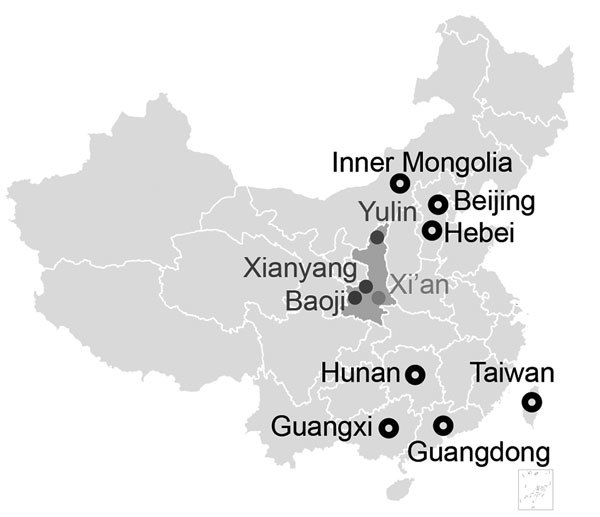
Geographic distribution of the avian influenza A(H7N9) viruses isolated in Shaanxi Province, China, 2016–2017 (solid circles), and of HPAI H7N9 viruses detected in other provinces of China (open circles).

We analyzed each of 8 gene segments of H7N9 viruses by using phylogenetic methods, together with available sequences from the GISAID EpiFlu database (http://platform.gisaid.org/; accession numbers in Technical Appendix Table 3). The HA and neuraminidase (NA) phylogenies indicate that the viruses isolated in Shaanxi belong to clade W2-C (*9*). In the HA tree (Figure 1; Technical Appendix Figure 1), the 16 HPAI H7N9 viruses we isolated clustered with previously detected HPAI H7N9 viruses from Guangdong Province. The HA gene of the Shaanxi HPAI H7N9 viruses has a common ancestor with the Guangdong HPAI H7N9 viruses, indicating that HPAI H7N9 viruses have spread from Guangdong to other provinces (Technical Appendix Figure 2, panel B). Further, the 7 LPAI H7N9 viruses we identified in Shaanxi Province clustered with LPAI H7N9 viruses isolated from various districts in eastern (Anhui, Fujian, Zhejiang, Jiangsu), central (Chongqing, Henan, Hunan, Hubei), and southern (Guangxi, Guangdong, Hong Kong) China, indicating that LPAI H7N9 viruses have been spreading from eastern and southern China into western China (Figure 2). In the NA phylogeny, the phylogenetic positions of the NA genes of all viruses we sequenced were similar to those observed in the HA phylogeny (Technical Appendix Figure 2). Phylogenies of the 6 internal genes (Technical Appendix Figure 3) show that the viruses from Shaanxi Province belong to clades 1 and 2 (*9*) of the ZJ-HJ/07 lineage of H9N2 viruses (*10*) (online Technical Appendix Table 2).

Phylogenetic analyses of HA and NA gene segments reveal that the viruses obtained from human cases in Yulin and Baoji were genetically similar to those from environmental samples collected from local farms or LPMs in Yulin and Baoji (Technical Appendix Figure 1, panel A; Technical Appendix Figure 2), suggesting that the human H7N9 infections are related to viruses circulating in local farms or LPMs. This hypothesis is also supported by most internal gene segments, except for the NS segments of viruses from Yulin (no internal gene segments were obtained for isolate A/Environment/Shaanxi/BJ2/2017 sampled in Baoji). Most of the gene segments of the viruses from human cases in Baoji and Xianyang were similar to those of A/Environment/Shaanxi/XA4/2017, the virus isolated from LPMs in Xi’an (with the exception of PB1 from the human case in Baoji and PB2, PB1, and matrix of the viruses from human cases in Xianyang). The NS and polymerase acidic segments of the LPAI virus from a human case in Xi’an (A/Shaanxi/XA1/2017) were genetically similar to the HPAI viruses we isolated; other gene segments were close to LPAI viruses from Anhui Province in southern China.

## Conclusions

We report emerging HPAI H7N9 variants in Shaanxi Province, western China. Our phylogenetic analyses support that the LPAI H7N9 viruses in Shaanxi Province originated from eastern and southern China, and the Shaanxi HPAI H7N9 isolates probably originated in Guangdong Province and were transmitted either directly or indirectly through other provinces.

Our results provide evidence that H7N9 viruses infecting humans in Shaanxi Province derived, directly or indirectly, from strains circulating in local farms and LPMs. Previous studies suggest that poultry trade between LPMs may play a key role in spreading H7N9 viruses (*11*). Similarly, we hypothesize that live poultry or poultry product transport may facilitate the spread of HPAI H7N9 viruses out of Guangdong Province and within Shaanxi Province.

Technical Appendix Environmental sample information, genotypes, accession numbers, and phylogenetic analysis of hemagglutinin sequences of avian influenza A(H7N9) viruses isolated in Shaanxi Province, China, and of reference viruses.

## References

[1] Gao R, Cao B, Hu Y, Feng Z, Wang D, Hu W, et al. Human infection with a novel avian-origin influenza A (H7N9) virus. N Engl J Med. 2013;368:1888–97. 10.1056/NEJMoa1304459 23577628

[2] World Health Organization. Monthly Risk Assessment Summary. Influenza at the human-animal interface. 2017 Oct 30 [cited 2017 Dec 7]. http://www.who.int/influenza/human_animal_interface/Influenza_Summary_IRA_HA_interface_10_30_2017.pdf

[3] Zhang F, Bi Y, Wang J, Wong G, Shi W, Hu F, et al. Human infections with recently-emerging highly pathogenic H7N9 avian influenza virus in China. J Infect. 2017;75:71–5. 10.1016/j.jinf.2017.04.001 28390707

[4] Zhu W, Zhou J, Li Z, Yang L, Li X, Huang W, et al. Biological characterisation of the emerged highly pathogenic avian influenza (HPAI) A(H7N9) viruses in humans, in mainland China, 2016 to 2017. Euro Surveill. 2017;22:30533. 10.2807/1560-7917.ES.2017.22.19.30533 28537546 PMC5476987

[5] Yang JR, Liu MT. Human infection caused by an avian influenza A (H7N9) virus with a polybasic cleavage site in Taiwan, 2017. J Formos Med Assoc. 2017;116:210–2. 10.1016/j.jfma.2017.02.011 28259506

[6] Ke C, Mok CKP, Zhu W, Zhou H, He J, Guan W, et al. Human infection with highly pathogenic avian influenza A(H7N9) virus, China. Emerg Infect Dis. 2017;23:1332–40. 10.3201/eid2308.170600 28580899 PMC5547808

[7] Shi Y, Zhang W, Wang F, Qi J, Wu Y, Song H, et al. Structures and receptor binding of hemagglutinins from human-infecting H7N9 influenza viruses. Science. 2013;342:243–7. 10.1126/science.1242917 24009358

[8] Zhou J, Wang D, Gao R, Zhao B, Song J, Qi X, et al. Biological features of novel avian influenza A (H7N9) virus. Nature. 2013;499:500–3. 10.1038/nature12379 23823727

[9] Lam TT, Zhou B, Wang J, Chai Y, Shen Y, Chen X, et al. Dissemination, divergence and establishment of H7N9 influenza viruses in China. Nature. 2015;522:102–5. 10.1038/nature14348 25762140

[10] Lam TT, Wang J, Shen Y, Zhou B, Duan L, Cheung C-L, et al. The genesis and source of the H7N9 influenza viruses causing human infections in China. Nature. 2013;502:241–4. 10.1038/nature12515 23965623 PMC3801098

[11] Zhou X, Li Y, Wang Y, Edwards J, Guo F, Clements ACA, et al. The role of live poultry movement and live bird market biosecurity in the epidemiology of influenza A (H7N9): A cross-sectional observational study in four eastern China provinces. J Infect. 2015;71:470–9. 10.1016/j.jinf.2015.06.012 26149187

