# Supplementary material for: Westward Spread of Highly Pathogenic Avian Influenza A(H7N9) Virus among Humans, China
Source: Emerg Infect Dis. 2018 Jun;24(6):1095–8. doi: 10.3201/eid2406.171135 (PMC6004833; doi:10.3201/eid2406.171135)
Supplement: Technical Appendix — Environmental sample information, genotypes, accession numbers, and phylogenetic analysis of hemagglutinin sequences of avian influenza A(H7N9) viruses isolated in Shaanxi Province, China, and of reference viruses. [file 17-1135-Techapp-s1.pdf]

Article DOI: https://doi.org/10.3201/eid2406.141135

Westward Spread of Highly Pathogenic Avian Influenza A(H7N9) Virus among Humans, China

Technical Appendix

Technical Appendix Table 1. Sampling information of all environmental samples.

City	Sample type	No. samples from farms	No. samples from LPMs
Baoji	Total	0	70
	H5-postive	0	1
	H7N9-positive	0	3
	H9-positive	0	4
Xi'an	Total	0	209
	H7N9-positive	0	11
Xianyang	Total	192	0
	H9-positive	5	0
Yulin	Total	25	0
	H7N9-positive	16	0
	H9-positive	1	0

Technical Appendix Table 2. Genotypes of all viruses newly isolated in Shaanxi Province in this study*

Sequence	HA	NA	PB2	PB1	PA	NP	M	NS
A/Shaanxi/XY1/2017	Clade W2-C	Clade W2-C	Clade 1	Clade 2	Clade 2	Clade 1	Clade 2	Clade 1
A/Shaanxi/XY2/2017	Clade W2-C	Clade W2-C				Clade 1	Clade 2	Clade 1
A/Shaanxi/BJ1/2017	Clade W2-C	Clade W2-C	Clade 1	Clade 1	Clade 2	Clade 1	Clade 2	Clade 1
A/Shaanxi/XA1/2017	Clade W2-C	Clade W2-C	Clade 1	Clade 1	Clade 1	Clade 1	Clade 2	Clade 1
A/Shaanxi/YL1/2017	Clade W2-C	Clade W2-C	Clade 2	Clade 1	Clade 1	Clade 1	Clade 2	Clade 1
A/Environment/Shaanxi/XA1/2017	Clade W2-C	Clade W2-C	Clade 2	Clade 1	Clade 1	Clade 1	Clade 2	Clade 1
A/Environment/Shaanxi/XA2/2017	Clade W2-C	Clade W2-C	Clade 2	Clade 1	Clade 1	Clade 1	Clade 2	Clade 1
A/Environment/Shaanxi/BJ1/2017	Clade W2-C	Clade W2-C	Clade 2	Clade 1	Clade 1	Clade 1	Clade 2	Clade 1
A/Environment/Shaanxi/BJ2/2017	Clade W2-C	Clade W2-C						
A/Environment/Shaanxi/YL1/2017	Clade W2-C							
A/Environment/Shaanxi/XA4/2017 <mixed>	Clade W2-C	Clade W2-C	Clade 1	Clade 1	Clade 2	Clade 1	Clade 2	Clade 1
A/Environment/Shaanxi/YL2/2017	Clade W2-C	Clade W2-C	Clade 2	Clade 1	Clade 1	Clade 1	Clade 2	Clade 1
A/Environment/Shaanxi/YL3/2017								Clade 1

Sequence	HA	NA	PB2	PB1	PA	NP	M	NS
A/Environment/Shaanxi/YL4/2017	Clade W2-C	Clade W2-C	Clade 2	Clade 1	Clade 1	Clade 1	Clade 2	Clade 1
A/Environment/Shaanxi/YL5/2017							Clade 2	
A/Environment/Shaanxi/YL6/2017	Clade W2-C	W2-C	Clade 2	Clade 1	Clade 1	Clade 1	Clade 2	Clade 1
A/Environment/Shaanxi/YL7/2017		Clade W2-C	Clade 2			Clade 1	Clade 2	Clade 1
A/Environment/Shaanxi/YL8/2017	Clade W2-C	Clade W2-C	Clade 2	Clade 1	Clade 1	Clade 1	Clade 2	Clade 1
A/Environment/Shaanxi/YL9/2017	Clade W2-C	Clade W2-C	Clade 2	Clade 1	Clade 1	Clade 1	Clade 2	Clade 1
A/Environment/Shaanxi/YL10/2017	Clade W2-C	Clade W2-C	Clade 2	Clade 1	Clade 1	Clade 1	Clade 2	Clade 1
A/Environment/Shaanxi/YL11/2017	Clade W2-C	Clade W2-C	Clade 2	Clade 1	Clade 1	Clade 1	Clade 2	Clade 1
A/Environment/Shaanxi/YL12/2017	Clade W2-C	Clade W2-C	Clade 2	Clade 1	Clade 1	Clade 1	Clade 2	Clade 1
A/Environment/Shaanxi/YL13/2017	Clade W2-C	Clade W2-C	Clade 2	Clade 1	Clade 1	Clade 1	Clade 2	Clade 1
A/Environment/Shaanxi/YL14/2017	Clade W2-C	Clade W2-C	Clade 2	Clade 1		Clade 1	Clade 2	Clade 1
A/Environment/Shaanxi/YL15/2017	Clade W2-C	Clade W2-C	Clade 2		Clade 1	Clade 1	Clade 2	Clade 1
A/Environment/Shaanxi/YL16/2017	Clade W2-C	Clade W2-C	Clade 2	Clade 1	Clade 1	Clade 1	Clade 2	Clade 1

*Blank cells indicate no nucleotide sequence was obtained. HA, hemagglutinin; NA, neuraminidase; PB, polybasic; PA, polymerase acidic; NP, nucleoprotein; M, matrix protein; NS, nonstructural protein. Abbreviations in the sequences indicate origins: XY, Xianyang; BJ, Baoj; XA, Xi'an; YL, Yulin.

Technical Appendix Table 3. Accession numbers of the GISAID and GenBank sequences used in this study.

Gene	Accession numbers
HA	EPI_ISL_267756, EPI_ISL_267757, EPI_ISL_267758, EPI_ISL_267759, EPI_ISL_267760, EPI_ISL_267761, EPI_ISL_267763, EPI_ISL_267764, EPI_ISL_268508, EPI_ISL_268512, EPI_ISL_268515, EPI_ISL_268523, EPI_ISL_268524, EPI_ISL_268525, EPI_ISL_269510, EPI_ISL_269511, EPI_ISL_269512, EPI_ISL_269513, EPI_ISL_269514, EPI_ISL_269515, EPI_ISL_269516, EPI_ISL_269517, EPI_ISL_269518, EPI_ISL_269519, EPI_ISL_269520, EPI_ISL_269521, EPI_ISL_269522, EPI_ISL_273350, EPI_ISL_273351, EPI_ISL_273353, EPI_ISL_273354, EPI_ISL_273944, EPI_ISL_273947, EPI_ISL_273948, EPI_ISL_273949, EPI_ISL_273950, EPI_ISL_273952, EPI_ISL_273953, EPI_ISL_277446, EPI_ISL_277447, EPI_ISL_277448, EPI_ISL_273302, EPI_ISL_277449, EPI_ISL_278766, EPI_ISL_278767, EPI_ISL_278769, EPI_ISL_278771, EPI_ISL_278772, EPI_ISL_278773, EPI_ISL_278775, EPI_ISL_278776, EPI_ISL_278777, EPI_ISL_278779, EPI_ISL_278780, EPI_ISL_278781, EPI_ISL_278782, EPI_ISL_278783, EPI_ISL_278784, EPI_ISL_278786, EPI_ISL_278788, EPI_ISL_278789, EPI_ISL_278790, EPI_ISL_278791, EPI_ISL_258409, EPI_ISL_138737, EPI_ISL_142915, EPI_ISL_141170, EPI_ISL_139499, EPI_ISL_142905, EPI_ISL_139905, EPI_ISL_175824, EPI_ISL_141159, EPI_ISL_142906, EPI_ISL_142922, EPI_ISL_153077, EPI_ISL_157293, EPI_ISL_160435, EPI_ISL_175633, EPI_ISL_175634, EPI_ISL_176442, EPI_ISL_175736, EPI_ISL_175787, EPI_ISL_180034, EPI_ISL_180096, EPI_ISL_180100, EPI_ISL_180141, EPI_ISL_180148, EPI_ISL_142916, EPI_ISL_146183, EPI_ISL_153006, EPI_ISL_156304, EPI_ISL_157151, EPI_ISL_159070, EPI_ISL_161665, EPI_ISL_161673, EPI_ISL_163320, EPI_ISL_172819, EPI_ISL_172822, EPI_ISL_192315, EPI_ISL_192430, EPI_ISL_176413, EPI_ISL_176835, EPI_ISL_176836, EPI_ISL_175650, EPI_ISL_175684, EPI_ISL_175712, EPI_ISL_175718, EPI_ISL_175737, EPI_ISL_175781, EPI_ISL_175789, EPI_ISL_176038, EPI_ISL_179851, EPI_ISL_179918, EPI_ISL_180077, EPI_ISL_180073, EPI_ISL_176165, EPI_ISL_176167, EPI_ISL_176169, EPI_ISL_176205, EPI_ISL_182439, EPI_ISL_190691, EPI_ISL_192274, EPI_ISL_192281, EPI_ISL_192283, EPI_ISL_192286, EPI_ISL_192478, EPI_ISL_192288, EPI_ISL_192293, EPI_ISL_192296, EPI_ISL_192300, EPI_ISL_192301, EPI_ISL_192307, EPI_ISL_192321, EPI_ISL_192329, EPI_ISL_192331, EPI_ISL_192340, EPI_ISL_192387, EPI_ISL_192395, EPI_ISL_192406, EPI_ISL_192413, EPI_ISL_192438, EPI_ISL_192453, EPI_ISL_192469, EPI_ISL_192470, EPI_ISL_192471, EPI_ISL_192472, EPI_ISL_192479, EPI_ISL_192499, EPI_ISL_192489, EPI_ISL_192490, EPI_ISL_192491, EPI_ISL_192493, EPI_ISL_192496, EPI_ISL_192501, EPI_ISL_205122, EPI_ISL_205126, EPI_ISL_206846, EPI_ISL_206851, EPI_ISL_206850, EPI_ISL_215688, EPI_ISL_217947, EPI_ISL_233628, EPI_ISL_239994, EPI_ISL_242275, EPI_ISL_242844, EPI_ISL_242846, EPI_ISL_242849, EPI_ISL_242851, EPI_ISL_258007, EPI_ISL_242867, EPI_ISL_242870, EPI_ISL_242883, EPI_ISL_242892, EPI_ISL_242895, EPI_ISL_242897, EPI_ISL_248778, EPI_ISL_249102, EPI_ISL_249309, EPI_ISL_249310, EPI_ISL_253626, EPI_ISL_256109, EPI_ISL_257983, EPI_ISL_257984, EPI_ISL_257986, EPI_ISL_257988,

Gene	Accession numbers
	EPI_ISL_257994, EPI_ISL_257996, EPI_ISL_257999, EPI_ISL_258003, EPI_ISL_258015, EPI_ISL_258029, EPI_ISL_258035, EPI_ISL_258245, EPI_ISL_263365, EPI_ISL_249308
NA	EPI_ISL_439487, EPI_ISL_439509, EPI_ISL_440096, EPI_ISL_583186, EPI_ISL_441797, EPI_ISL_442714, EPI_ISL_447716, EPI_ISL_447632, EPI_ISL_447653, EPI_ISL_447681, EPI_ISL_447730, EPI_ISL_447737, EPI_ISL_447787, EPI_ISL_447797, EPI_ISL_447829, EPI_ISL_447884, EPI_ISL_477309, EPI_ISL_453606, EPI_ISL_453611, EPI_ISL_457628, EPI_ISL_457700, EPI_ISL_457716, EPI_ISL_457724, EPI_ISL_457732, EPI_ISL_457764, EPI_ISL_457772, EPI_ISL_457788, EPI_ISL_457796, EPI_ISL_457836, EPI_ISL_457860, EPI_ISL_497859, EPI_ISL_457884, EPI_ISL_467338, EPI_ISL_470349, EPI_ISL_477404, EPI_ISL_490970, EPI_ISL_497831, EPI_ISL_497838, EPI_ISL_497851, EPI_ISL_498089, EPI_ISL_498100, EPI_ISL_506235, EPI_ISL_507149, EPI_ISL_509122, EPI_ISL_509887, EPI_ISL_516605, EPI_ISL_521916, EPI_ISL_521919, EPI_ISL_528297, EPI_ISL_531470, EPI_ISL_566059, EPI_ISL_566083, EPI_ISL_577899, EPI_ISL_578003, EPI_ISL_578017, EPI_ISL_578031, EPI_ISL_597441, EPI_ISL_578187, EPI_ISL_580373, EPI_ISL_580381, EPI_ISL_581604, EPI_ISL_581712, EPI_ISL_581726, EPI_ISL_581746, EPI_ISL_581853, EPI_ISL_581860, EPI_ISL_581967, EPI_ISL_582349, EPI_ISL_582939, EPI_ISL_583104, EPI_ISL_583124, EPI_ISL_583941, EPI_ISL_592026, EPI_ISL_592110, EPI_ISL_592654, EPI_ISL_592246, EPI_ISL_593383, EPI_ISL_592276, EPI_ISL_592601, EPI_ISL_593148, EPI_ISL_593162, EPI_ISL_593169, EPI_ISL_593197, EPI_ISL_593204, EPI_ISL_593296, EPI_ISL_593321, EPI_ISL_593336, EPI_ISL_596935, EPI_ISL_593407, EPI_ISL_593471, EPI_ISL_593543, EPI_ISL_596826, EPI_ISL_596909, EPI_ISL_596915, EPI_ISL_596942, EPI_ISL_596949, EPI_ISL_596956, EPI_ISL_597155, EPI_ISL_597162, EPI_ISL_597305, EPI_ISL_597448, EPI_ISL_621121, EPI_ISL_626984, EPI_ISL_627000, EPI_ISL_627040, EPI_ISL_627056, EPI_ISL_627080, EPI_ISL_627096, EPI_ISL_627136, EPI_ISL_627160, EPI_ISL_627192, EPI_ISL_627200, EPI_ISL_627248, EPI_ISL_627312, EPI_ISL_627360, EPI_ISL_627424, EPI_ISL_627440, EPI_ISL_627887, EPI_ISL_628095, EPI_ISL_628415, EPI_ISL_628551, EPI_ISL_628559, EPI_ISL_628567, EPI_ISL_628615, EPI_ISL_628623, EPI_ISL_628695, EPI_ISL_628703, EPI_ISL_628711, EPI_ISL_628719, EPI_ISL_628735, EPI_ISL_628783, EPI_ISL_628799, EPI_ISL_683027, EPI_ISL_683030, EPI_ISL_692613, EPI_ISL_692618, EPI_ISL_692619, EPI_ISL_730494, EPI_ISL_740737, EPI_ISL_833707, EPI_ISL_869672, EPI_ISL_884221, EPI_ISL_887643, EPI_ISL_971147, EPI_ISL_971291, EPI_ISL_887667, EPI_ISL_887683, EPI_ISL_887811, EPI_ISL_887835, EPI_ISL_887939, EPI_ISL_888011, EPI_ISL_888027, EPI_ISL_888035, EPI_ISL_917067, EPI_ISL_918738, EPI_ISL_919598, EPI_ISL_919606, EPI_ISL_960365, EPI_ISL_919614, EPI_ISL_945070, EPI_ISL_971099, EPI_ISL_971107, EPI_ISL_971123, EPI_ISL_971131, EPI_ISL_971139, EPI_ISL_971187, EPI_ISL_971203, EPI_ISL_971227, EPI_ISL_971355, EPI_ISL_971427, EPI_ISL_971467, EPI_ISL_971515, EPI_ISL_972305, EPI_ISL_973365, EPI_ISL_997219, EPI_ISL_1010185, EPI_ISL_267751, EPI_ISL_267756, EPI_ISL_267757, EPI_ISL_267758, EPI_ISL_267760, EPI_ISL_267761, EPI_ISL_267763, EPI_ISL_267764, EPI_ISL_268498, EPI_ISL_268499, EPI_ISL_268500, EPI_ISL_268503, EPI_ISL_268505, EPI_ISL_268506, EPI_ISL_268507, EPI_ISL_268508, EPI_ISL_268509, EPI_ISL_268512, EPI_ISL_268523, EPI_ISL_268524, EPI_ISL_268525, EPI_ISL_268526, EPI_ISL_269510, EPI_ISL_269512, EPI_ISL_269513, EPI_ISL_269514, EPI_ISL_269515, EPI_ISL_269516, EPI_ISL_269517, EPI_ISL_269518, EPI_ISL_269519, EPI_ISL_269520, EPI_ISL_269521, EPI_ISL_269522, EPI_ISL_273350, EPI_ISL_273351, EPI_ISL_273352, EPI_ISL_273353, EPI_ISL_273354, EPI_ISL_273848, EPI_ISL_273943, EPI_ISL_273944, EPI_ISL_273947, EPI_ISL_273948, EPI_ISL_273949, EPI_ISL_273950, EPI_ISL_273952, EPI_ISL_273953, EPI_ISL_277446, EPI_ISL_277447, EPI_ISL_277448, EPI_ISL_273302, EPI_ISL_277449, EPI_ISL_283474, EPI_ISL_283475, EPI_ISL_283476, EPI_ISL_283479, EPI_ISL_283480, EPI_ISL_283481, EPI_ISL_283482, EPI_ISL_283485, EPI_ISL_283486, EPI_ISL_283487, EPI_ISL_283489, EPI_ISL_283492, EPI_ISL_283494, EPI_ISL_283495, EPI_ISL_283496, EPI_ISL_283500, EPI_ISL_283501, EPI_ISL_283504, EPI_ISL_283505, EPI_ISL_283506, EPI_ISL_283507, EPI_ISL_283509, EPI_ISL_283510, EPI_ISL_283512, EPI_ISL_283513, EPI_ISL_283514, EPI_ISL_283515, EPI_ISL_283516, EPI_ISL_283519, EPI_ISL_283520, EPI_ISL_283521, EPI_ISL_283522, EPI_ISL_283523, EPI_ISL_283525, EPI_ISL_283526, EPI_ISL_283527, EPI_ISL_283530, EPI_ISL_283531, EPI_ISL_283535, EPI_ISL_283540, EPI_ISL_283541, EPI_ISL_283542, EPI_ISL_283543, EPI_ISL_283546, EPI_ISL_283549, EPI_ISL_283552, EPI_ISL_283553, EPI_ISL_283555
PB2 (33% H9N2, 67% H7N9)	EPI_ISL_258010, EPI_ISL_242883, EPI_ISL_242861, EPI_ISL_729800, EPI_ISL_269517, EPI_ISL_257986, EPI_ISL_283496, EPI_ISL_257989, EPI_ISL_242901, EPI_ISL_192469, EPI_ISL_192471, EPI_ISL_242862, EPI_ISL_242881, EPI_ISL_257988, EPI_ISL_258007, EPI_ISL_242843, EPI_ISL_242890, EPI_ISL_212471, EPI_ISL_942257, EPI_ISL_683192, EPI_ISL_269515, EPI_ISL_258035, EPI_ISL_258028, EPI_ISL_273302, EPI_ISL_258009, EPI_ISL_283495, EPI_ISL_242845, EPI_ISL_239994, EPI_ISL_257994, EPI_ISL_192478, EPI_ISL_599387, EPI_ISL_192490, EPI_ISL_192300, EPI_ISL_599430, EPI_ISL_192477, EPI_ISL_172824, EPI_ISL_192502, EPI_ISL_141165, EPI_ISL_141166, EPI_ISL_141166, EPI_ISL_175604, EPI_ISL_146186, EPI_ISL_142916, EPI_ISL_192451, EPI_ISL_194370, EPI_ISL_192489, EPI_ISL_223673, EPI_ISL_162874, EPI_ISL_142905, EPI_ISL_192395, EPI_ISL_176207, EPI_ISL_176171, EPI_ISL_283482, EPI_ISL_175642, EPI_ISL_192429,

Gene	Accession numbers
	EPI_ISL_180073, EPI_ISL_176169, EPI_ISL_283491, EPI_ISL_283493, EPI_ISL_269521, EPI_ISL_268504, EPI_ISL_269514, EPI_ISL_269520, EPI_ISL_269519, EPI_ISL_269518, EPI_ISL_269522, EPI_ISL_269516, EPI_ISL_268523, EPI_ISL_268512, EPI_ISL_269510, EPI_ISL_273950, EPI_ISL_269512, EPI_ISL_267755, EPI_ISL_283542, EPI_ISL_242866, EPI_ISL_258011, EPI_ISL_233629, EPI_ISL_248778, EPI_ISL_729799, EPI_ISL_180021, EPI_ISL_192301, EPI_ISL_681056, EPI_ISL_175612, EPI_ISL_161674, EPI_ISL_192308, EPI_ISL_172820, EPI_ISL_160435, EPI_ISL_175597, EPI_ISL_192424, EPI_ISL_166324, EPI_ISL_192412, EPI_ISL_141170, EPI_ISL_176116, EPI_ISL_192325, EPI_ISL_192351, EPI_ISL_161665, EPI_ISL_179966, EPI_ISL_192294, EPI_ISL_283508, EPI_ISL_173693, EPI_ISL_157293, FJ581429, JF519817, JF795141, JQ356886, JN653566, CY087176, JN653567, JN869514, JF519809, JN869521, JQ356884, JN869535, JQ356884, KC417067, KC464595, JN222387, FJ793385, AY253750, CY055153, EU753319, JF795043, GQ202061, DQ064550, EU935070, AF523471, FJ793345, CY023637, CY023733, CY023725, CY041273, CY043863, AB473937, JN543666, JX097043, CY081259, JF745928, EF063555, CY038431, JX437691, HQ221632, EF154849, AF156435, JQ901631, CY023805, AF508654, AF508656, AF156436, AF508650, AF536681
PB1<br>(25% H9N2, 75% H7N9)	EPI_ISL_140356, EPI_ISL_139499, EPI_ISL_141183, EPI_ISL_141179, EPI_ISL_141159, EPI_ISL_141158, EPI_ISL_141776, EPI_ISL_142916, EPI_ISL_142922, EPI_ISL_142923, EPI_ISL_146186, EPI_ISL_146876, EPI_ISL_148727, EPI_ISL_151417, EPI_ISL_153011, EPI_ISL_154827, EPI_ISL_161670, EPI_ISL_161675, EPI_ISL_162470, EPI_ISL_163998, EPI_ISL_166324, EPI_ISL_172827, EPI_ISL_175604, EPI_ISL_175641, EPI_ISL_176439, EPI_ISL_176442, EPI_ISL_176417, EPI_ISL_176820, EPI_ISL_175649, EPI_ISL_175737, EPI_ISL_175792, EPI_ISL_179859, EPI_ISL_180078, EPI_ISL_180088, EPI_ISL_180103, EPI_ISL_176114, EPI_ISL_176196, EPI_ISL_176203, EPI_ISL_176207, EPI_ISL_176285, EPI_ISL_192287, EPI_ISL_192289, EPI_ISL_192297, EPI_ISL_192301, EPI_ISL_192335, EPI_ISL_192356, EPI_ISL_192378, EPI_ISL_192387, EPI_ISL_192412, EPI_ISL_192440, EPI_ISL_192470, EPI_ISL_192471, EPI_ISL_192482, EPI_ISL_192485, EPI_ISL_192493, EPI_ISL_194370, EPI_ISL_205123, EPI_ISL_205125, EPI_ISL_206707, EPI_ISL_212471, EPI_ISL_215688, EPI_ISL_217947, EPI_ISL_233629, EPI_ISL_240829, EPI_ISL_242847, EPI_ISL_242853, EPI_ISL_242854, EPI_ISL_242856, EPI_ISL_242865, EPI_ISL_242866, EPI_ISL_242869, EPI_ISL_242873, EPI_ISL_242882, EPI_ISL_242885, EPI_ISL_242890, EPI_ISL_242891, EPI_ISL_242897, EPI_ISL_242901, EPI_ISL_244621, EPI_ISL_248778, EPI_ISL_249102, EPI_ISL_252837, EPI_ISL_257981, EPI_ISL_257983, EPI_ISL_257987, EPI_ISL_257990, EPI_ISL_257994, EPI_ISL_257995, EPI_ISL_258008, EPI_ISL_258010, EPI_ISL_258014, EPI_ISL_258017, EPI_ISL_258028, EPI_ISL_267755, EPI_ISL_267759, EPI_ISL_267761, EPI_ISL_267764, EPI_ISL_268506, EPI_ISL_268507, EPI_ISL_268508, EPI_ISL_268512, EPI_ISL_268525, EPI_ISL_269510, EPI_ISL_269511, EPI_ISL_269514, EPI_ISL_269516, EPI_ISL_269517, EPI_ISL_269518, EPI_ISL_269519, EPI_ISL_269520, EPI_ISL_269521, EPI_ISL_269522, EPI_ISL_273350, EPI_ISL_273950, EPI_ISL_277046, EPI_ISL_277048, EPI_ISL_277050, EPI_ISL_273302, EPI_ISL_283473, EPI_ISL_283475, EPI_ISL_283477, EPI_ISL_283480, EPI_ISL_283493, EPI_ISL_283496, EPI_ISL_283502, EPI_ISL_283505, EPI_ISL_283507, EPI_ISL_283511, EPI_ISL_283515, EPI_ISL_283521, EPI_ISL_283522, EPI_ISL_283526, EPI_ISL_283532, EPI_ISL_283533, EPI_ISL_283539, EPI_ISL_283541, EPI_ISL_283542, EPI_ISL_283546, EPI_ISL_283553, EPI_ISL_283552, EPI_ISL_578197, EPI_ISL_583690, EPI_ISL_583739, EPI_ISL_683148, EPI_ISL_838012, EPI_ISL_844496, EPI_ISL_844500, EPI_ISL_846037, EPI_ISL_942235, EPI_ISL_1034489, AF508632, AF156422, AY253751, AF508634, AF523442, CY043862, CY055154, AF156421, KC464596, JN222386, JN653582, EF063534, CY023638, AF508628, DQ064523, JN869536, CY023734, CY023726, GQ202060, FJ793346, FJ492968, CY023806, JX097036, FJ793386, JN543644, CY038432, CY081261, JF795142, JF795044, EU753320, KC417064, CY087177, JQ356887, JQ356889, FJ581435, JN869522, AB473938, HQ221704, JX437686, JQ901620, JF745929, CY041272, EF155360
PA<br>(32% H9N2, 68% H7N9)	EPI_ISL_138983, EPI_ISL_139498, EPI_ISL_141188, EPI_ISL_141171, EPI_ISL_142231, EPI_ISL_142858, EPI_ISL_142920, EPI_ISL_142921, EPI_ISL_142922, EPI_ISL_142923, EPI_ISL_145654, EPI_ISL_146185, EPI_ISL_153013, EPI_ISL_153077, EPI_ISL_154828, EPI_ISL_156304, EPI_ISL_157284, EPI_ISL_159478, EPI_ISL_161672, EPI_ISL_162874, EPI_ISL_164002, EPI_ISL_164003, EPI_ISL_164006, EPI_ISL_166324, EPI_ISL_169442, EPI_ISL_172823, EPI_ISL_172825, EPI_ISL_172829, EPI_ISL_173693, EPI_ISL_175596, EPI_ISL_175679, EPI_ISL_175709, EPI_ISL_176043, EPI_ISL_176186, EPI_ISL_176204, EPI_ISL_176835, EPI_ISL_179919, EPI_ISL_180025, EPI_ISL_180088, EPI_ISL_180090, EPI_ISL_190692, EPI_ISL_192278, EPI_ISL_192282, EPI_ISL_192283, EPI_ISL_192285, EPI_ISL_192294, EPI_ISL_192307, EPI_ISL_192311, EPI_ISL_192322, EPI_ISL_192329, EPI_ISL_192357, EPI_ISL_192367, EPI_ISL_192382, EPI_ISL_192384, EPI_ISL_192386, EPI_ISL_192396, EPI_ISL_192399, EPI_ISL_192402, EPI_ISL_192405, EPI_ISL_192412, EPI_ISL_192433, EPI_ISL_192450, EPI_ISL_192452, EPI_ISL_192469, EPI_ISL_192471, EPI_ISL_192478, EPI_ISL_192495, EPI_ISL_192497, EPI_ISL_192499, EPI_ISL_192501, EPI_ISL_267759, EPI_ISL_267761, EPI_ISL_268498, EPI_ISL_268505, EPI_ISL_268507, EPI_ISL_268508, EPI_ISL_268526, EPI_ISL_269510, EPI_ISL_269512, EPI_ISL_269514, EPI_ISL_269517, EPI_ISL_269518, EPI_ISL_269519, EPI_ISL_269520, EPI_ISL_269521,

Gene	Accession numbers
	EPI_ISL_269522, EPI_ISL_273950, EPI_ISL_273952, EPI_ISL_277048, EPI_ISL_273301, EPI_ISL_283480, EPI_ISL_283486, EPI_ISL_283493, EPI_ISL_283505, EPI_ISL_283507, EPI_ISL_283514, EPI_ISL_283518, EPI_ISL_283521, EPI_ISL_283525, EPI_ISL_283527, EPI_ISL_283528, EPI_ISL_283551, EPI_ISL_529225, EPI_ISL_681080, EPI_ISL_683235, EPI_ISL_838060, EPI_ISL_1034481, AF508676, AF156450, AY253752, AF508678, CY043861, CY055155, AF156449, KC464597, JN222380, JN653598, EF063548, CY023639, AF508672, DQ064496, JQ639785, JN869537, CY023735, CY023727, GQ202063, FJ793347, JN653599, CY023807, JX097029, JQ254979, FJ793387, JN543622, JN543617, HQ221588, JN852800, CY038433, CY081263, JF795143, JF795045, JQ904461, EU753321, KC417061, CY024607, CY023263, CY087178, JQ356883, FJ581430, JN869523, AB473939, JQ901642, JF745930, CY041271, JN828569, EF155287
NP<br>(28% H9N2, 72% H7N9)	EPI_ISL_141185, EPI_ISL_141171, EPI_ISL_142187, EPI_ISL_142306, EPI_ISL_142905, EPI_ISL_142912, EPI_ISL_142921, EPI_ISL_142923, EPI_ISL_146369, EPI_ISL_154827, EPI_ISL_156527, EPI_ISL_157293, EPI_ISL_162618, EPI_ISL_164002, EPI_ISL_155815, EPI_ISL_172823, EPI_ISL_175607, EPI_ISL_175637, EPI_ISL_176431, EPI_ISL_175648, EPI_ISL_175683, EPI_ISL_175732, EPI_ISL_175778, EPI_ISL_179964, EPI_ISL_179982, EPI_ISL_176204, EPI_ISL_176280, EPI_ISL_176282, EPI_ISL_176305, EPI_ISL_176364, EPI_ISL_192278, EPI_ISL_192279, EPI_ISL_192298, EPI_ISL_192384, EPI_ISL_192390, EPI_ISL_192463, EPI_ISL_192468, EPI_ISL_192469, EPI_ISL_192475, EPI_ISL_192501, EPI_ISL_194307, EPI_ISL_194386, EPI_ISL_194388, EPI_ISL_205128, EPI_ISL_206749, EPI_ISL_217947, EPI_ISL_242275, EPI_ISL_242843, EPI_ISL_242865, EPI_ISL_242873, EPI_ISL_242887, EPI_ISL_242890, EPI_ISL_248778, EPI_ISL_249308, EPI_ISL_249309, EPI_ISL_249310, EPI_ISL_256108, EPI_ISL_256109, EPI_ISL_257986, EPI_ISL_257988, EPI_ISL_258007, EPI_ISL_258014, EPI_ISL_258027, EPI_ISL_258409, EPI_ISL_263365, EPI_ISL_267757, EPI_ISL_267758, EPI_ISL_267761, EPI_ISL_267762, EPI_ISL_267763, EPI_ISL_267764, EPI_ISL_268499, EPI_ISL_268500, EPI_ISL_268501, EPI_ISL_268503, EPI_ISL_268506, EPI_ISL_268507, EPI_ISL_268509, EPI_ISL_268512, EPI_ISL_268515, EPI_ISL_268523, EPI_ISL_268524, EPI_ISL_269510, EPI_ISL_269512, EPI_ISL_269514, EPI_ISL_269516, EPI_ISL_269518, EPI_ISL_269519, EPI_ISL_269520, EPI_ISL_269521, EPI_ISL_269522, EPI_ISL_273350, EPI_ISL_273950, EPI_ISL_273952, EPI_ISL_277047, EPI_ISL_277447, EPI_ISL_283497, EPI_ISL_283505, EPI_ISL_283520, EPI_ISL_283521, EPI_ISL_283523, EPI_ISL_283524, EPI_ISL_599552, EPI_ISL_681125, EPI_ISL_846032, AF508610, AF156408, AY253753, AF508612, AF523414, CY043859, CY055157, AF156407, KC464599, JN653630, EF063527, CY023641, AF508606, DQ064438, DQ064442, JN869539, CY023737, CY023729, GQ202062, FJ793349, FJ492973, JN653631, CY023809, FJ793389, JN543594, CY038435, CY120060, GU050557, JF795145, JF795047, EU753323, JF519795, CY087180, JQ356878, JQ356880, FJ581428, JN869524, AB473940, HQ221722, JX437689, JQ901664, JF745932, CY041269, EF155141
M<br>(29% H9N2, 71% H7N9)	EPI_ISL_139649, EPI_ISL_139908, EPI_ISL_141182, EPI_ISL_141160, EPI_ISL_142902, EPI_ISL_142905, EPI_ISL_142908, EPI_ISL_143663, EPI_ISL_146876, EPI_ISL_148299, EPI_ISL_151429, EPI_ISL_157287, EPI_ISL_162470, EPI_ISL_169442, EPI_ISL_172824, EPI_ISL_173033, EPI_ISL_175608, EPI_ISL_175642, EPI_ISL_176417, EPI_ISL_175646, EPI_ISL_175718, EPI_ISL_175726, EPI_ISL_175792, EPI_ISL_179977, EPI_ISL_180023, EPI_ISL_180028, EPI_ISL_176050, EPI_ISL_176132, EPI_ISL_176196, EPI_ISL_176373, EPI_ISL_192283, EPI_ISL_192284, EPI_ISL_192293, EPI_ISL_192298, EPI_ISL_192299, EPI_ISL_192307, EPI_ISL_192308, EPI_ISL_192309, EPI_ISL_192323, EPI_ISL_192333, EPI_ISL_192339, EPI_ISL_192341, EPI_ISL_192344, EPI_ISL_192352, EPI_ISL_192364, EPI_ISL_192392, EPI_ISL_192408, EPI_ISL_192410, EPI_ISL_192435, EPI_ISL_192469, EPI_ISL_192490, EPI_ISL_192499, EPI_ISL_192504, EPI_ISL_194993, EPI_ISL_205124, EPI_ISL_215533, EPI_ISL_239994, EPI_ISL_242275, EPI_ISL_242846, EPI_ISL_242862, EPI_ISL_242875, EPI_ISL_242890, EPI_ISL_257986, EPI_ISL_257988, EPI_ISL_257995, EPI_ISL_258005, EPI_ISL_258007, EPI_ISL_258008, EPI_ISL_258009, EPI_ISL_258018, EPI_ISL_258021, EPI_ISL_258028, EPI_ISL_258035, EPI_ISL_258409, EPI_ISL_267755, EPI_ISL_267759, EPI_ISL_268515, EPI_ISL_268523, EPI_ISL_268525, EPI_ISL_269510, EPI_ISL_269512, EPI_ISL_269516, EPI_ISL_269518, EPI_ISL_269519, EPI_ISL_269520, EPI_ISL_269521, EPI_ISL_269522, EPI_ISL_273353, EPI_ISL_273354, EPI_ISL_273950, EPI_ISL_277046, EPI_ISL_277050, EPI_ISL_277450, EPI_ISL_283475, EPI_ISL_283477, EPI_ISL_283480, EPI_ISL_283496, EPI_ISL_283505, EPI_ISL_283520, EPI_ISL_283532, EPI_ISL_283553, EPI_ISL_469974, EPI_ISL_583328, EPI_ISL_682977, EPI_ISL_682980, EPI_ISL_729782, EPI_ISL_838159, AF508697, AF156464, AY253755, AF508699, AF523493, CY043857, CY055159, AF156463, KC464601, JN222383, JN653662, AF536721, EF063506, CY023643, JN869519, AF508694, DQ064388, JN869541, CY023739, CY023731, GQ202059, FJ793351, EU835749, JN653663, CY023811, FJ793391, CY038437, CY120061, JF795147, JF795049, EU753325, KC417049, CY087182, JQ356890, JQ356892, FJ581432, JN869526, AB473941, HQ221650, JX437684, JQ901686, JF745933, CY041267, EF154995
NS<br>(28% H9N2, 72% H7N9)	EPI_ISL_141177, EPI_ISL_141173, EPI_ISL_141172, EPI_ISL_142859, EPI_ISL_142905, EPI_ISL_142906, EPI_ISL_142908, EPI_ISL_148417, EPI_ISL_159478, EPI_ISL_172818, EPI_ISL_172821, EPI_ISL_172823, EPI_ISL_173693, EPI_ISL_175713, EPI_ISL_175724, EPI_ISL_169131, EPI_ISL_179898, EPI_ISL_179910, EPI_ISL_180073, EPI_ISL_176168,

Gene	Accession numbers
	EPI_ISL_176290, EPI_ISL_192280, EPI_ISL_192300, EPI_ISL_192323, EPI_ISL_192331, EPI_ISL_192340, EPI_ISL_192422, EPI_ISL_192427, EPI_ISL_192450, EPI_ISL_192453, EPI_ISL_192470, EPI_ISL_192472, EPI_ISL_192479, EPI_ISL_192493, EPI_ISL_192501, EPI_ISL_192505, EPI_ISL_212471, EPI_ISL_220945, EPI_ISL_233628, EPI_ISL_240829, EPI_ISL_242275, EPI_ISL_242843, EPI_ISL_242845, EPI_ISL_242846, EPI_ISL_242859, EPI_ISL_242864, EPI_ISL_242870, EPI_ISL_242874, EPI_ISL_242887, EPI_ISL_242891, EPI_ISL_249102, EPI_ISL_257986, EPI_ISL_257988, EPI_ISL_258008, EPI_ISL_258010, EPI_ISL_258012, EPI_ISL_258023, EPI_ISL_258024, EPI_ISL_258027, EPI_ISL_258032, EPI_ISL_258409, EPI_ISL_267753, EPI_ISL_267762, EPI_ISL_268501, EPI_ISL_268512, EPI_ISL_268523, EPI_ISL_269510, EPI_ISL_269512, EPI_ISL_269513, EPI_ISL_269515, EPI_ISL_269516, EPI_ISL_269518, EPI_ISL_269519, EPI_ISL_269520, EPI_ISL_269521, EPI_ISL_269522, EPI_ISL_273351, EPI_ISL_273353, EPI_ISL_273952, EPI_ISL_277446, EPI_ISL_283495, EPI_ISL_283505, EPI_ISL_283508, EPI_ISL_283511, EPI_ISL_283513, EPI_ISL_283518, EPI_ISL_283521, EPI_ISL_283522, EPI_ISL_283523, EPI_ISL_283531, EPI_ISL_283532, EPI_ISL_283533, EPI_ISL_283541, EPI_ISL_283552, EPI_ISL_838182, EPI_ISL_838196, EPI_ISL_846043, EPI_ISL_942297, EPI_ISL_AF508719, EPI_ISL_AF156478, EPI_ISL_AY253756, EPI_ISL_AF508721, CY055160, AF156477, KC464602, JN653678, AF536731, EF063541, CY023644, AF508715, DQ064469, JN869542, CY023740, CY023732, GQ202058, FJ793352, EU835745, JN653679, CY023812, FJ793392, JN543560, CY038438, CY081268, JF795148, JF795050, EU753326, KC417058, CY087183, JQ356893, JQ356895, FJ581433, JN869527, AB473942, HQ221614, JQ901697, JF745934, CY041270, EF155214

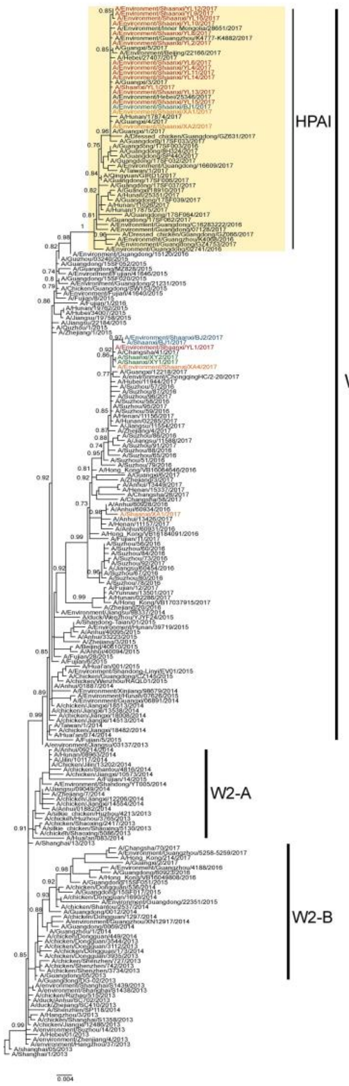

Technical Appendix Figure 1. A) Phylogenetic analysis of HA sequences of avian influenza A (H7N9) viruses isolated in Shaanxi province, China, together with reference viruses. Sequence name colors represent the sampling location of the H7N9 viruses obtained in this study: Yulin (red), Xianyang (green), Baoji (blue), and Xi’an (orange). The clade highlighted in yellow contains previously isolated HPAI A (H7N9) viruses. Maximum likelihood phylogenies were estimated by using a GTR+gamma nucleotide substitution model, with Shimodaira-Hasegawa-like local-bootstrap support values for node support (*1*), as implemented in PhyML v3.1 (*2*). B) Detail of the HPAI sub-clade highlighted in panel A, with SH-like local-bootstrap support values. Amino acid changes within the HA cleavage site are indicated on basal branches. The LPAI strain A/Environment/Guangdong/15120/2016 is used as an outgroup.

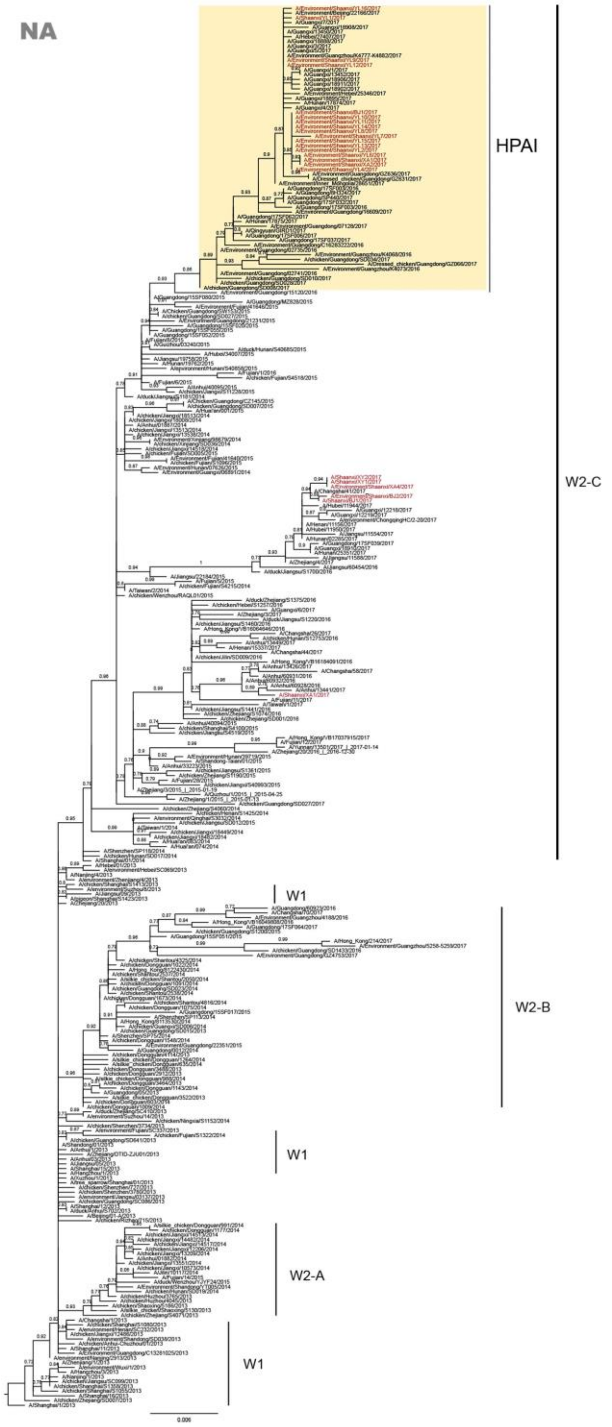

Technical Appendix Figure 2. Maximum likelihood phylogenies of the neuraminidase (NA) gene segments. This tree was inferred from NA gene sequences. Taxa colored red are the sequences isolated in this study. Taxa highlighted in yellow are previously isolated highly pathogenic avian influenza (HPAI) A (H7N9) viruses.

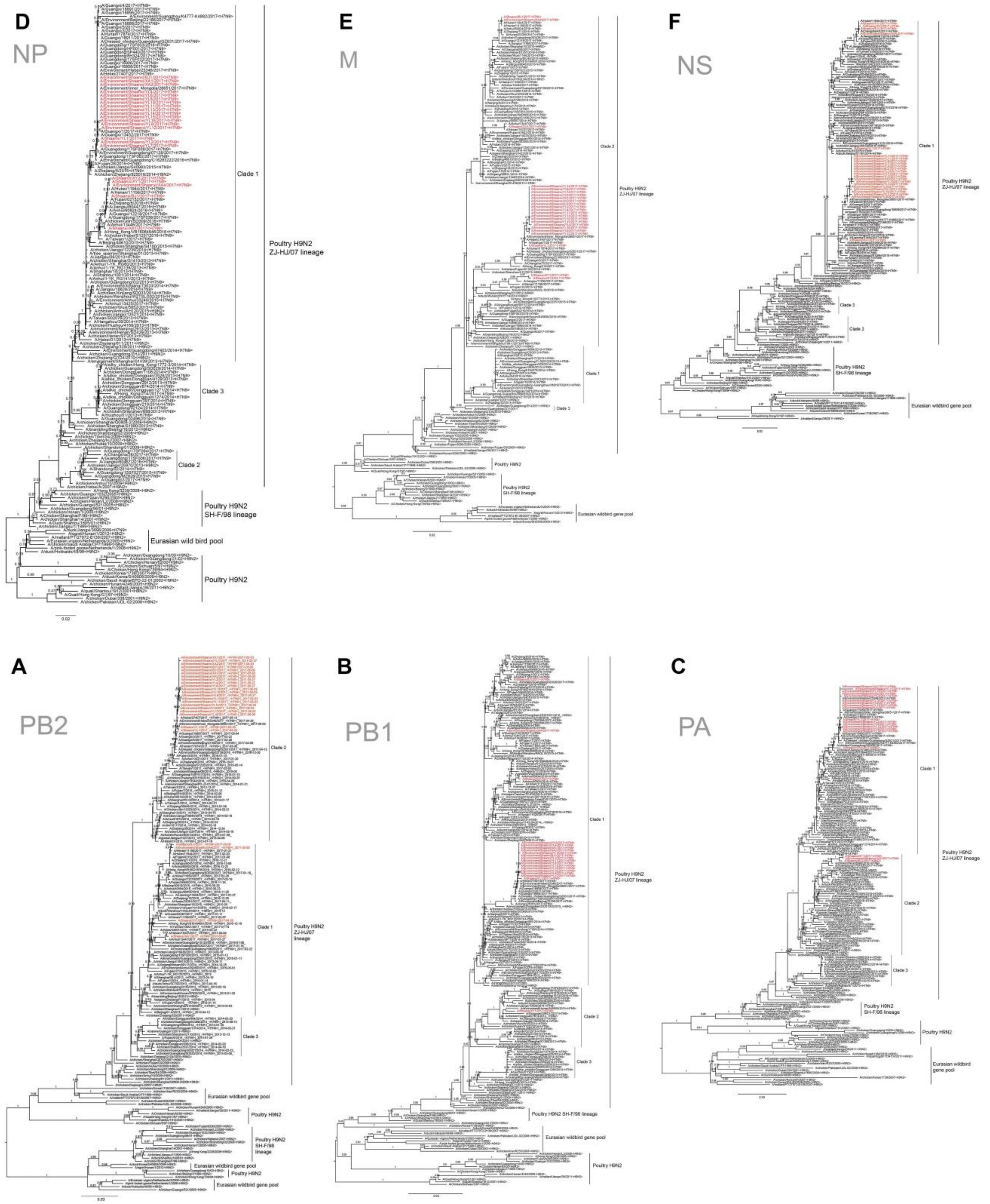

Technical Appendix Figure 3. A) Maximum likelihood phylogenies of the internal gene segments. This tree was inferred from PB2 gene sequences. Sequences isolated in this study are colored red.

Shimodaira-Hasegawa-like branch support values are shown. Major clades 1–3 of ZJ-HJ/07 lineage of H9N2 viruses are labeled. Poultry H9N2, Poultry H9N2 SH-F/98 lineage and Eurasian wildbird gene pool are labeled as outgroups. **B)** Maximum likelihood phylogenies of the internal gene segments. This tree was inferred from PB1 gene sequences. Sequences isolated in this study are colored red. Shimodaira-Hasegawa-like branch support values are shown. Major clades 1–3 of ZJ-HJ/07 lineage of H9N2 viruses are labeled. Poultry H9N2, Poultry H9N2 SH-F/98 lineage and Eurasian wildbird gene pool are labeled as outgroups. **C)** Maximum likelihood phylogenies of the internal gene segments. This tree was inferred from PA gene sequences. Sequences isolated in this study are colored red. Shimodaira-Hasegawa-like branch support values are shown. Major clades 1–3 of ZJ-HJ/07 lineage of H9N2 viruses are labeled. Poultry H9N2, Poultry H9N2 SH-F/98 lineage and Eurasian wildbird gene pool are labeled as outgroups. **D)** Maximum likelihood phylogenies of the internal gene segments. This tree was inferred from NP gene sequences. Sequences isolated in this study are colored red. Shimodaira-Hasegawa-like branch support values are shown. Major clades 1–3 of ZJ-HJ/07 lineage of H9N2 viruses are labeled. Poultry H9N2, Poultry H9N2 SH-F/98 lineage and Eurasian wildbird gene pool are labeled as outgroups. **E).** Maximum likelihood phylogenies of the internal gene segments. This tree was inferred from M gene sequences. Sequences isolated in this study are colored red. Shimodaira-Hasegawa-like branch support values are shown. Major clades 1–3 of ZJ-HJ/07 lineage of H9N2 viruses are labeled. Poultry H9N2, Poultry H9N2 SH-F/98 lineage and Eurasian wildbird gene pool are labeled as outgroups. **F)** Maximum likelihood phylogenies of the internal gene segments. This tree was inferred from NS gene sequences. Sequences isolated in this study are colored red. Shimodaira-Hasegawa-like branch support values are shown. Major clades 1–3 of ZJ-HJ/07 lineage of H9N2 viruses are labeled. Sequences belonging to Eurasian wildbird gene pool, Poultry H9N2 and Poultry H9N2 SH-F/98 lineage are labeled as outgroups.

References

1. Guindon S, Dufayard J-F, Lefort V, Anisimova M, Hordijk W, Gascuel O. New algorithms and methods to estimate maximum-likelihood phylogenies: assessing the performance of PhyML 3.0. Syst Biol. 2010;59:307–21. PubMed http://dx.doi.org/10.1093/sysbio/syq010

2. Guindon S, Gascuel O. A simple, fast, and accurate algorithm to estimate large phylogenies by maximum likelihood. Syst Biol. 2003;52:696–704. PubMed http://dx.doi.org/10.1080/10635150390235520
